# SARS CoV-2 seroprevalence and rising antibody titres across successive pandemic waves in Accra, Ghana: August 2020 to January 2022

**DOI:** 10.1038/s41598-026-57566-3

**Published:** 2026-06-22

**Authors:** P. J. Pappoe-Ashong, J. A. A. Mingle, D. Tetteh, C. A. Brown, S. K. Nordzi, B. T. Mensah, J. A. Oliver-Commey, P. Puplampu, C. Jassoy

**Affiliations:** 1 Department of Medical Microbiology, University of Ghana Medical School, Accra, Ghana; 2 Institute of Medical Microbiology and Virology, University Clinics and Medical Faculty, University of Leipzig, Leipzig, Germany; 3 College of Health Sciences, School of Biomedical and Allied Health Sciences, Department of Medical Laboratory Sciences, University of Ghana, Accra, Ghana; 4 Greater Accra Regional Hospital Laboratory, Accra, Ghana; 5 Ghana Infectious Disease Centre, Accra, Ghana; 6 College of Health Sciences, School of Medicine, Department of Medicine, University of Ghana, Accra, Ghana

**Keywords:** Seroprevalence, Antibody titres, Exposure, SARS CoV-2, Ghana, Sub-Saharan Africa, Diseases, Immunology, Medical research, Microbiology

## Abstract

Shortly after its first outbreak in Wuhan, the SARS CoV-2 spread worldwide. This study determined SARS CoV-2 seroprevalence and compared anti-RBD IgG titre distributions among seropositive samples across four repeated cross-sectional surveys conducted during successive epidemic waves from August 2020 to January 2022. Age-specific seroprevalence and titre patterns were also examined across children, adolescents, and adults. We conducted a seroepidemiological cross-sectional study over four epidemic waves periods. A stratified 1,096 samples, reflecting the age and sex distribution of the Accra population, was selected from 15,000 residual clinical samples. Adjusted seroprevalence increased from 58.3% (95% CI: 53.9–62.6%) in wave 1 to > 90% by wave 2 and remained ≥ 96% in waves 3 and 4. Adults aged ≥ 20 years reached near-universal seropositivity earlier than children, who accumulated exposure more gradually. Despite this early plateau, geometric mean anti-RBD IgG concentrations increased from 162 BAU/mL in wave 1 to 625 BAU/mL in wave 3, stabilising at 584 BAU/mL in wave 4. Analyses of antibody titre distributions demonstrated that progressively larger proportions of adults showed concentrations substantially exceeding wave 1 baseline levels in later waves, indicating age-related differences in cumulative antigenic exposure. Across successive epidemic waves, Accra experienced intense and repeated SARS CoV-2 transmission, resulting in near-universal exposure of the adult population by May 2021. Children and adolescents showed a slower and more gradual increase in both seroprevalence and antibody concentrations. Anti-RBD IgG titres increased across successive waves and stabilised after August–November 2021, consistent with sustained cumulative antigenic exposure at the population level.

**Supplementary Information:**

The online version contains supplementary material available at 10.1038/s41598-026-57566-3.

## Introduction

 The emergence of the severe acute respiratory syndrome coronavirus 2 (SARS CoV-2) in late 2019 led to a global pandemic that strained health systems worldwide. In sub-Saharan Africa, the true burden of infection was difficult to quantify due to limited diagnostic capacity, high levels of asymptomatic infection, and gaps in surveillance infrastructure^1–6^. Ghana reported its first confirmed cases in March 2020, with the Greater Accra Region rapidly becoming the epicentre^7^. By June 2020, over 17,000 cases had been reported nationally, more than 60% of which were asymptomatic^8^. Approximately two-thirds of cases were recorded in Accra, and many were detected only through contact tracing and targeted community testing, indicating that case counts underestimated the true extent of exposure^9^.

Seroprevalence studies can capture both symptomatic and asymptomatic infections, though the sensitivity of detection varies with assay characteristics and the timing and magnitude of the antibody response^10^. Early serosurveys in Africa revealed widespread undetected SARS CoV-2 transmission^11^. In Ghana, a household study reported 67.10% seroprevalence between February and December 2021^12^. In Greater Accra, seroprevalence increased from 13.8% to 39.6% between November 2020 and July 2021^13^. Additional studies in blood donors and special populations reported comparably high exposure levels^14,15^.

The Greater Accra Region, with its high population density, was selected to examine patterns of SARS CoV-2 exposure across successive waves of infection. The aim of this study was to determine SARS CoV-2 seroprevalence and, among seropositive individuals, compare anti-RBD IgG titre distributions across four repeated cross-sectional surveys conducted during successive epidemic waves from August 2020 to January 2022. We further examined age-specific seroprevalence and titre distributions to characterise serological profiles among children, adolescents, and adults.

## Methodology

### Study design and setting

We conducted a repeated cross-sectional seroepidemiological study using anonymised residual serum and plasma samples collected at the following three hospitals in Accra. Ussher Hospital (UH), a municipal facility serving the high-density Ga Mashie catchment area in central Accra. Greater Accra Regional Hospital (GARH), located in Accra, the capital of both the Greater Accra Region and Ghana, serves as the main referral centre for the region. It draws patients from all districts of Greater Accra and, occasionally, from neighbouring regions including Eastern, Central, and Volta. Princess Marie Louise Children’s Hospital (PMLH), the only public paediatric hospital in Accra that serves children across multiple districts. Together, these facilities provide access to a demographically and socioeconomically diverse population in Greater Accra region enhancing the representativeness of the sampled population.

### Sample collection, stratification and selection

A total of 15,601 residual serum and plasma samples (in EDTA or serum separator tubes) were retrieved from routine clinical biochemistry and haematology laboratories, encompassing a broad range of clinical indications unrelated to SARS CoV-2 or respiratory illness between May 2020 and September 2022. Importantly, none of the samples included in this study were obtained from COVID-19–designated clinical pathways at any point during the study period. To minimise duplicate sampling, hospitals generated anonymised patient codes from available identifiers (patient ID, date of birth, sex). Only one sample per code was retained, and raw identifiers were not shared with the study team. All samples were anonymised at source, transferred to the testing facility, and stored at − 20 °C until serological testing. Samples that were haemolysed, icteric, lipaemic were excluded.

To enhance population representativeness, stratified random sampling was performed for each wave, matching age and sex distributions to the 2020 Accra Census^16^. Specimens drawn at the peak and decline of the incidence (supported by national case count figures) were selected for the analysis. Sampling periods and sample sizes were: wave 1, Aug–Oct 2020, *N* = 499, wave 2, Feb–May 2021, *N* = 203, wave 3, Aug–Nov 2021, *N* = 203, wave 4, Jan 2022, *N* = 193.

### Serological testing

In wave 1, an initial lateral flow immunoassay (LFA) screen was employed for operational reasons during the early epidemic response, with a subset of samples subsequently tested by ELISA to obtain quantitative titres and support serostatus classification. Samples were first screened using the Sero NP/RBD LFA, a qualitative assay detecting IgG against nucleocapsid (NP) and receptor-binding domain (RBD) antigens. This assay, previously validated in Ghana, demonstrated 96.1% sensitivity and 99.0% specificity^17^. Results were independently read by two trained personnel, with a third adjudicating discordant readings. Wave 1 seroprevalence estimates were derived from LFA results for all 499 samples, with targeted ELISA testing (*n* = 469) used to obtain quantitative anti-RBD IgG titres and to confirm the seronegative status of subset of LFA-negative samples. Samples reclassified as seropositive on ELISA were incorporated into the final wave 1 seroprevalence estimate, which is therefore reported as an adjusted measure integrating both LFA screening and confirmatory ELISA results. The same ELISA-tested subset was used to define baseline antibody titre distributions for wave 1. For waves 2–4, all selected samples were tested directly using the in-house anti-RBD IgG ELISA for both seropositivity determination and quantitative titre measurement. The assay was internally validated using 608 Ghanaian clinical sera (308 PCR-confirmed SARS CoV-2 positives and 300 pre-pandemic negatives), achieving > 97% diagnostic sensitivity and specificity at a cut-off of ≥ 32 BAU/mL.

### Quantitative anti-RBD IgG titre measurement

To determine anti-RBD IgG titres in serum and plasma samples, high-binding 96-well microtiter plates (Greiner BioOne) were coated with 1 µg/mL RBD (source of the protein^18^ in phosphate-buffered saline (PBS, pH 7.4) and incubated overnight at 4 °C. All subsequent steps were performed at room temperature. Plates were washed with deionised water and washing buffer (PBS with 0.05% Tween-20) and blocked with PBS containing 0.05% Tween-20 and 5% milk powder for 20 min. A serum standard calibrated to 250 BAU/mL using the Abbott IgG assay was included for RBD IgG quantification. Plasma or serum samples were diluted 1:100 in blocking solution and incubated for 60 min. After washing, goat anti-human IgG-HRP (Jackson ImmunoResearch, Art. No. 109-036-098) was added at 1:20,000 for RBD assays and incubated for 60 min. Plates were washed again, and 3,3′,5,5′-tetramethylbenzidine (TMB) substrate (SeramunBlue slow 2/85, Seramun Diagnostica GmbH) was added. The reaction was stopped after 15 min with 1 N sulphuric acid, and optical density (OD) was measured at 450 nm with a 630 nm reference wavelength. Samples exceeding the upper threshold OD were further diluted and re-analysed to ensure accurate quantification. Intra- and inter-assay reproducibility were assessed across five independent ELISA plates spanning 250 to 1.95 BAU/mL; inter-plate coefficients of variation were low at higher concentrations and remained within acceptable limits down to approximately 3.9 BAU/mL. The analytical limit of detection was below 32 BAU/mL; quantitative interpretation was conservatively restricted to concentrations ≥ 32 BAU/mL, supported by WHO calibration, internal validation, and demonstrated reproducibility. Positive and negative controls were included on every plate. No statistically significant differences in anti-RBD IgG titres were observed between serum and plasma matrices (venous serum median 804.7 BAU/mL vs. venous plasma 803.5 BAU/mL; all *p* > 0.05 (data not shown)), with strong Spearman correlations between venous and capillary matrices (ρ = 0.878 and 0.852, both *p* < 0.001) and negligible Bland-Altman bias on the log10 scale. Titres were expressed in binding antibody units per millilitre (BAU/mL), traceable to WHO International Standards, and seropositivity was defined as titres ≥ 32 BAU/mL, based on a validated threshold.

### Assessment of antibody titre distribution across epidemic waves

Quantitative anti-SARS CoV-2 RBD IgG concentrations were analysed across epidemic periods to characterise population-level antibody titre distributions across waves and age groups. Only samples with antibody concentrations ≥ 32 BAU/mL were included in titre analyses; seronegative samples were excluded from quantitative comparisons but are noted in figures to represent population-level distributions. Age-specific baseline geometric mean titres (GMTs) were defined using seropositive samples from August–October 2020 (wave 1). The proportions of samples in subsequent waves exceeding ≥ 4-fold and ≥ 8-fold multiples of the age-specific wave 1 GMT were calculated as operational indicators of population-level antibody titre accumulation, reflecting distributional shifts in antibody concentrations across waves rather than within-individual changes.

### Statistical analysis

Data were managed in Microsoft Excel 2021 and analysed in R version 4.5.1 using dplyr, tidyr, ggplot2, patchwork and readxl packages. Only samples with antibody concentrations ≥ 32 BAU/mL were included in quantitative titre analyses. Seronegative samples were excluded because antibody concentrations below the assay threshold cannot be reliably quantified; however, their frequency is captured in seroprevalence estimates and incorporated into the overall population-level interpretation. Seropositivity estimates with exact binomial 95% confidence intervals (Clopper–Pearson method) were calculated overall and stratified by age group. Anti-RBD IgG geometric mean titres were calculated using log-transformed data. Mann–Whitney U tests were used to assess differences in GMT distributions between waves. For threshold exceedance analyses, the proportions of samples exceeding ≥ 4-fold and ≥ 8-fold multiples of age-specific wave 1 GMTs were calculated for each age group and epidemic wave. Group differences in these proportions were assessed using Fisher’s exact tests with pairwise comparisons between age groups. To account for multiple comparisons, a Bonferroni-corrected significance threshold of *p* < 0.00238 (α = 0.05 ÷ 21) was applied, where 21 represents the number of pairwise age-group comparisons per wave and threshold. For GMT comparisons between waves (Mann–Whitney U tests), statistical significance was set at *p* < 0.05. For fold-threshold proportion comparisons (Fisher’s exact tests), raw p-values below the Bonferroni-corrected threshold are reported in Supplementary Table S4. Distribution of titres across waves and age groups was visualised using boxplots on a log scale with individual data points overlaid. All visualisations were generated using ggplot2.

**Fig. 1:**
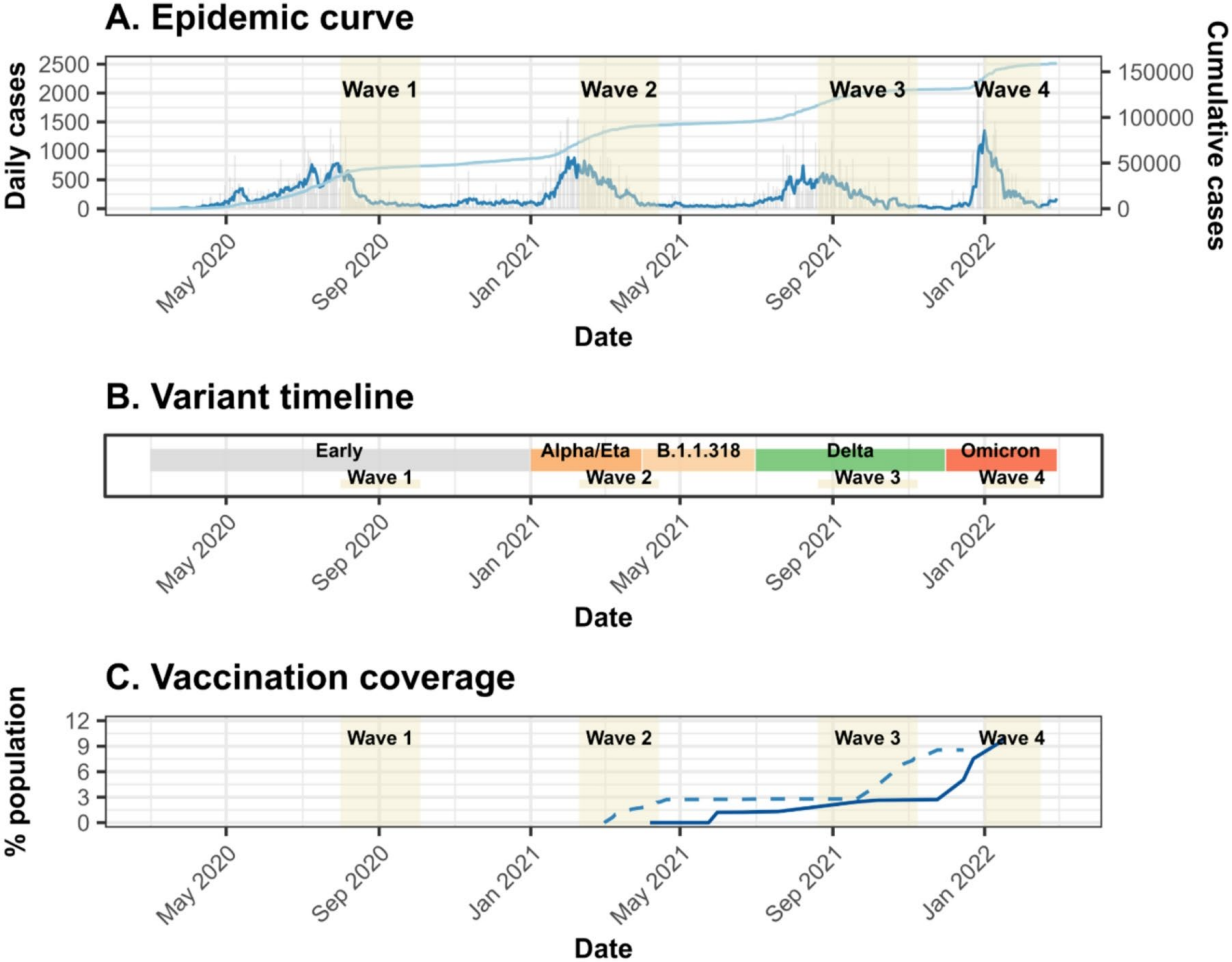
Epidemiological context of SARS CoV-2 transmission and study sampling in Accra, Ghana. **(A)** National epidemic curve with study sampling periods. Daily confirmed COVID-19 cases in Ghana (grey bars) and 7-day rolling average (dark blue line). Light blue line represents cumulative cases. Yellow shaded regions indicate the four wave sampling periods: wave 1, wave 2, wave 3, and wave 4. **(B)** Temporal distribution of predominant SARS CoV-2 variants. Timeline showing succession of predominant viral lineages in Ghana based on genomic surveillance. **(C)** Population vaccination coverage during study period. Blue line represents percentage of Ghana’s population fully vaccinated (2 doses) based on Our World in Data. Light blue line dashes ≥ 1 dose. National vaccination rollout began March 1, 2021. Data sources: Johns Hopkins University COVID-19 Data Repository (cases); Our World in Data (vaccination); published genomic surveillance reports in Ghana (variants)^19–21^.

**Fig. 2:**
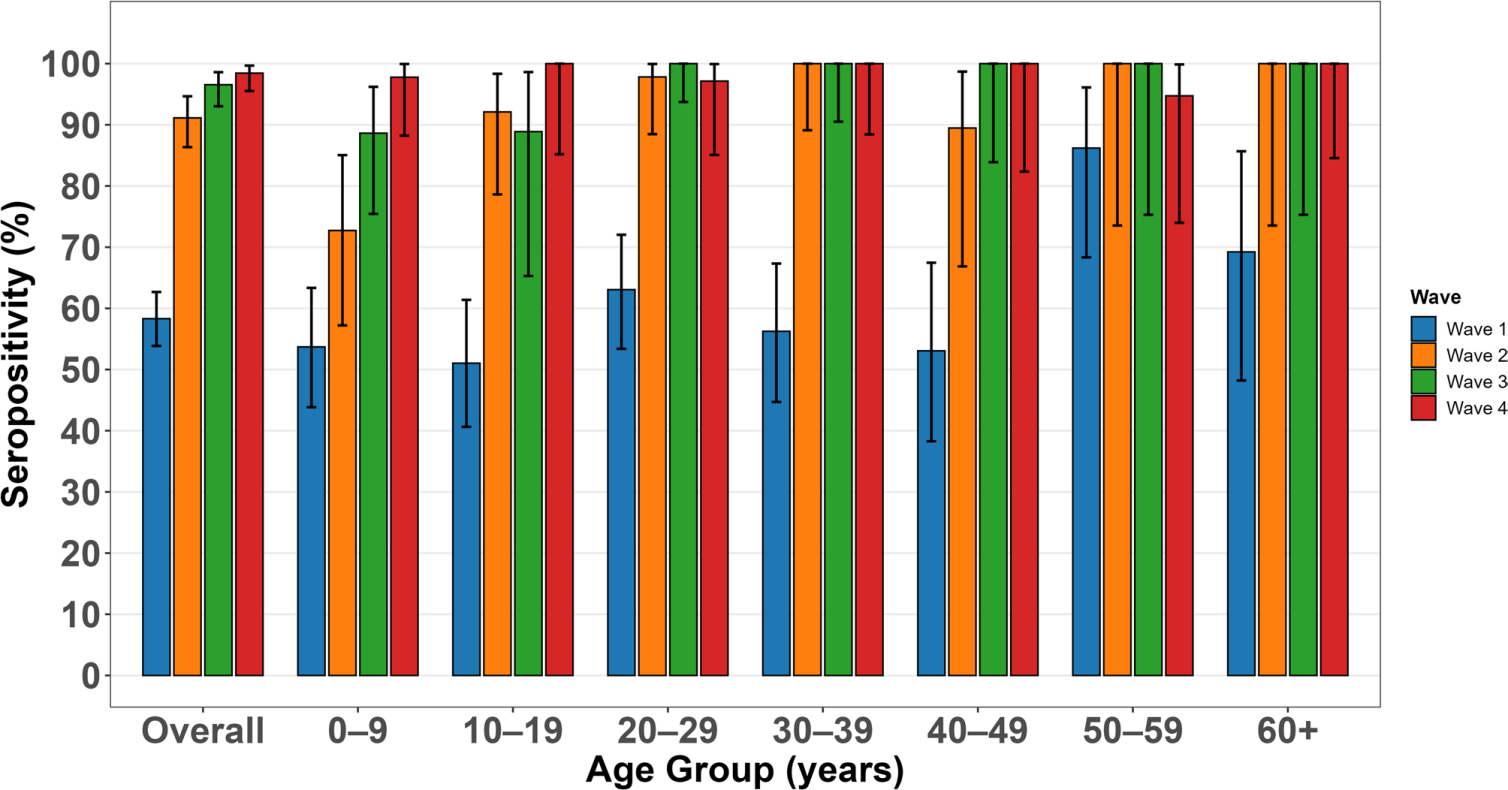
SARS CoV-2 IgG seropositivity by age group across four epidemic waves in Accra, Ghana. Seropositivity was defined as anti-RBD IgG concentrations ≥ 32 BAU/mL, measured using an in-house ELISA calibrated to WHO international standards. Estimates are shown with exact binomial 95% confidence intervals (Clopper–Pearson method) and are stratified by age group. Sample sizes per wave were: wave 1 (August–October 2020), *N* = 499; wave 2 (February–May 2021), *N* = 203; wave 3 (August–November 2021), *N* = 203; and wave 4 (January 2022), *N* = 193. Age-specific sample sizes are provided in Supplementary Table 1.

**Fig. 3:**
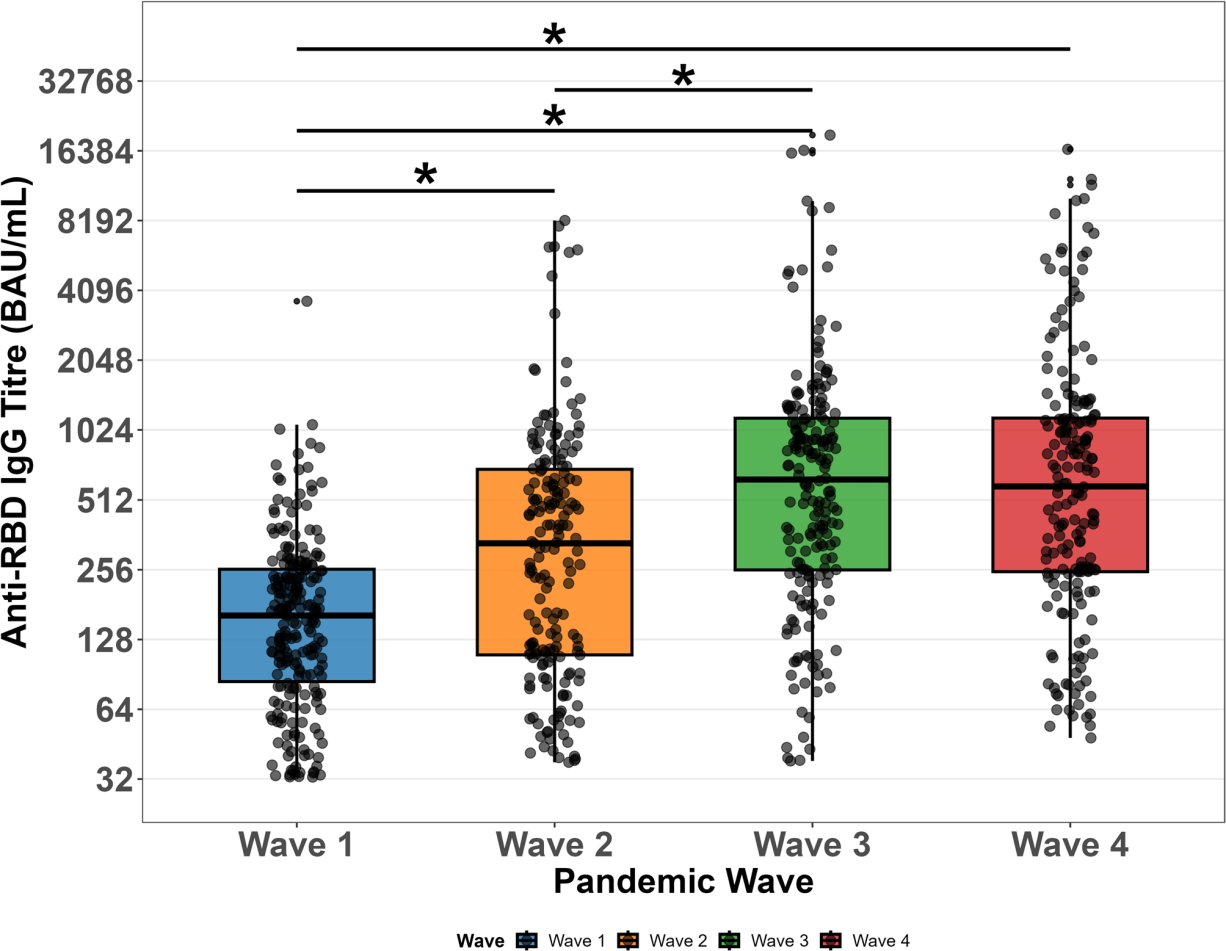
Distribution of individual anti-RBD IgG titres (BAU/mL) across waves. Differences in titre across waves with statistically significant differences in GMT between waves indicated by horizontal bars with asterisks (*p* < 0.05; Mann–Whitney U test). Only seropositive samples (≥ 32 BAU/mL) are included.

**Fig. 4:**
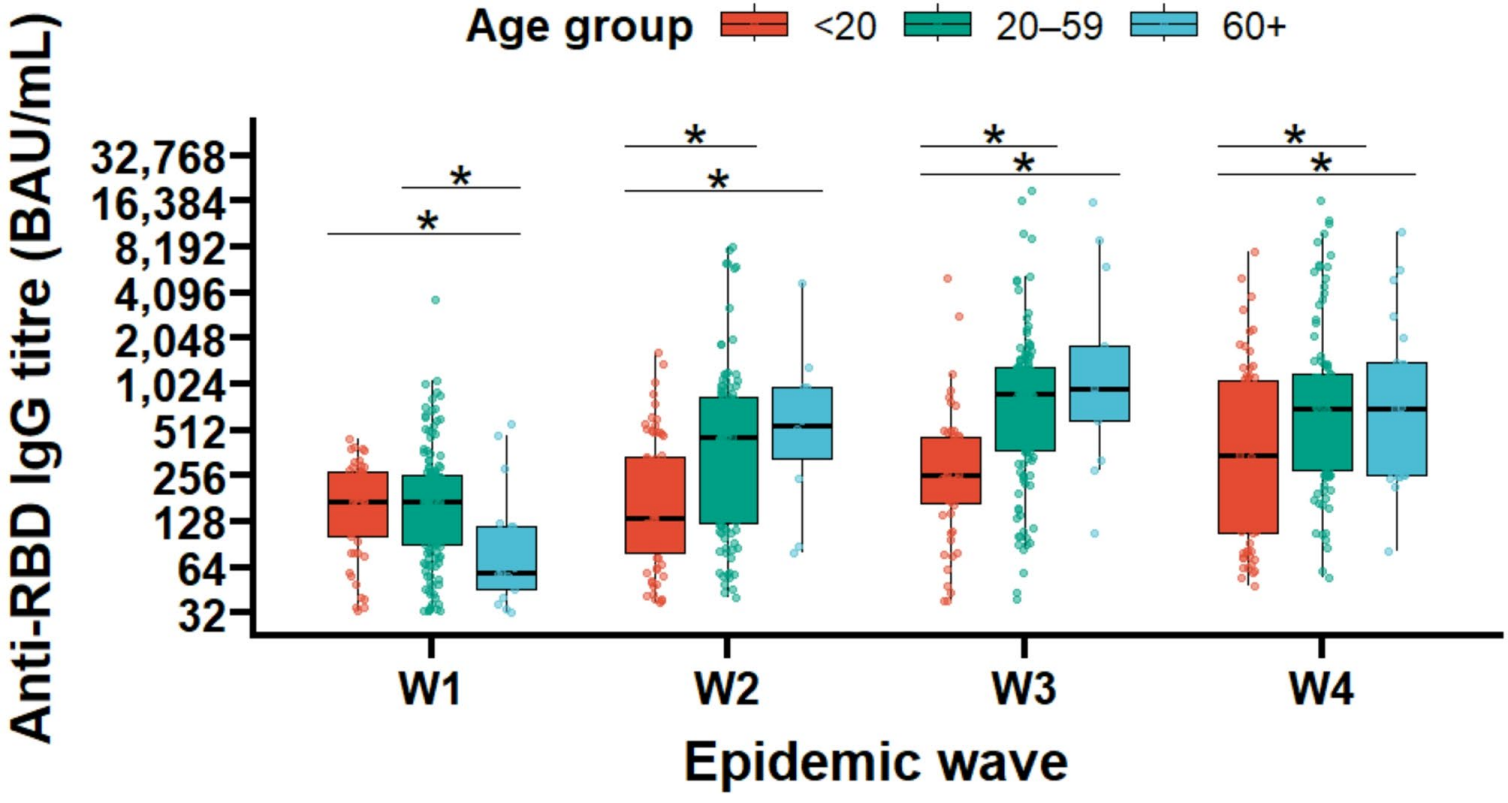
Distribution of anti-RBD IgG titres (BAU/mL) across epidemic waves, stratified by age group (< 20, 20–59, ≥ 60 years) in Accra, Ghana. Antibody concentrations are presented on a log scale. Boxes represent the interquartile range (IQR), centre lines indicate medians, and whiskers extend to 1.5× IQR. Individual data points are overlaid. Statistical differences between age groups within each wave are indicated by horizontal bars with asterisks (*p* < 0.05). W = epidemic wave. Only seropositive samples (≥ 32 BAU/mL) are included.

**Fig. 5:**
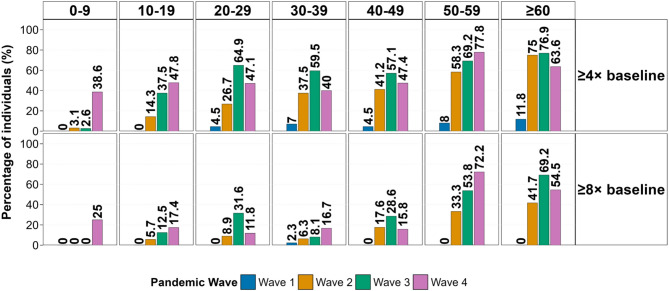
Proportion of seropositive individuals with anti-RBD IgG titres ≥ 4-fold and ≥ 8-fold above the age-specific wave 1 geometric mean titre across four epidemic waves. Bars represent age-stratified percentages for each wave. Wave 1 values reflect the proportion of wave 1 seropositive individuals whose titres exceeded these thresholds due to natural variation in antibody concentrations around the geometric mean and serve as the within-wave baseline reference. These thresholds represent population-level distributional comparisons across waves and should not be interpreted as within-individual fold changes.

## Results

### Overall and age-stratified anti-SARS CoV-2 IgG Seropositivity

Overall anti–SARS-CoV-2 IgG seroprevalence increased markedly across the four epidemic waves. Adjusted seroprevalence rose from 58.3% (95% CI: 53.9–62.6%) in Wave 1 (August–October 2020) to 91.1% (95% CI: 86.4–94.3%) in Wave 2 (February–May 2021), and remained high in Wave 3 (96.6%, 95% CI: 93.1–98.3%) and Wave 4 (98.5%, 95% CI: 95.5–99.5%). The largest increase occurred between Waves 1 and 2, after which seroprevalence plateaued at near-universal levels.

Age-stratified analyses showed that adults aged ≥ 20 years reached near-universal seropositivity earlier than younger age groups. By Wave 2, seroprevalence exceeded 97% across all adult age groups aged 20 years and above. In contrast, children aged 0–9 years and adolescents aged 10–19 years exhibited lower seroprevalence during the earlier waves, with progressive increases over time before converging with adult levels by Waves 3 and 4. By Wave 4, seroprevalence reached 97.8% among children aged 0–9 years and 100% among adolescents aged 10–19 years. Detailed age-stratified seroprevalence estimates, confidence intervals, and subgroup sample sizes are presented in Supplementary Table S1 and Fig. 2.

### Overall and age-related SARS CoV-2 anti-RBD IgG titre distribution

Anti-RBD IgG concentrations were approximately log-normally distributed at all time points. Geometric mean anti-RBD IgG titres (GMTs) increased from 162 BAU/mL in wave 1 to 411 BAU/mL in wave 2 and 625 BAU/mL in wave 3, then stabilised at 584 BAU/mL in wave 4. The increases between waves 1 and 2 and between waves 2 and 3 were statistically significant (*p* < 0.001), while titres stabilised between waves 3 and 4, consistent with an equilibrium between antibody waning and ongoing antigenic re-exposure (Fig. 2).

In the first wave, a slightly lower anti-RBD IgG concentration was observed in the group of adults above 60 years compared with adults 20–59 years and children and adolescents < 20 years (GMT: 84.7 vs. 148 and 161 BAU/ml, respectively, *p* < 0.05). In subsequent waves the antibody concentrations in sera from children and adolescents were consistently lower than in sera from 20 to 59 year and above 60 year old subjects (Fig. 3 and Supplementary Table S2 and S3).

### Age-stratified fold increases in anti-RBD IgG titres across epidemic waves

To characterise population-level shifts in antibody titre distributions, we calculated the proportions of samples in each wave whose anti-RBD IgG concentration exceeded ≥ 4-fold and ≥ 8-fold multiples of the age-specific wave 1 GMT. These thresholds are operational indicators of the magnitude of population-level antibody accumulation across waves, reflecting distributional shifts rather than within-individual changes. In wave 1, proportions meeting either threshold were low across all age groups, consistent with primary exposure.

In wave 2, ≥ 4-fold threshold exceedances were observed in at least 26.7% of participants aged ≥ 20 years (range: 26.7%–75.0%). Children aged 0–9 years exhibited significantly lower proportions (3.1%) than adults aged 30–39, 40–49, 50–59, and ≥ 60 years (all *p* ≤ 0.0014). Adolescents aged 10–19 years also showed lower proportions than adults aged ≥ 60 years (14.3% vs. 75.0%; raw p-value = 0.0002). For ≥ 8-fold threshold exceedances, proportions of 33.3% and 41.7% were observed among participants aged 50–59 and ≥ 60 years, respectively, with a significant difference detected only between children aged 0–9 years and adults aged ≥ 60 years (0.0% vs. 41.7%; p-value = 0.0007).

During waves 3 and 4, ≥ 4-fold threshold exceedances were observed in at least 37.5% of participants across most age groups, with the exception of children aged 0–9 years, among whom this threshold was reached only in wave 4 (38.6%). In wave 3, children aged 0–9 years differed significantly from all other age groups for ≥ 4-fold increases (all *p* ≤ 0.0016). For ≥ 8-fold increases, lower proportions among children aged 0–9 years compared with adults aged 20–29, 40–49, 50–59, and ≥ 60 years were observed (all *p* ≤ 0.0011). Additional differences were identified between adults aged 30–39 years and those aged 50–59 years (8.1% vs. 53.8%; *p* = 0.0014) and ≥ 60 years (8.1% vs. 69.2%; *p* < 0.0001). In wave 4, high titre accumulation (≥ 8-fold threshold exceedance) was most frequent among adults aged 50–59 years (72.2%). This group differed significantly from all younger age groups (all *p* ≤ 0.0012), and adults aged ≥ 60 years also differed from those aged 20–29 years (54.5% vs. 11.8%; *p* = 0.0008) (Fig. 5; Supplementary Table S4).

## Discussion

This repeated cross-sectional seroepidemiological study provides insight into the evolving landscape of SARS CoV-2 exposure in Accra across four successive epidemic waves. By integrating quantitative anti-RBD IgG measurements with seroprevalence, we show that although population seropositivity approached saturation early, antibody concentrations continued to rise, indicating ongoing exposure beyond what could be inferred from binary serostatus alone.

The rapid rise in seropositivity between waves 1 and 2, followed by persistently high levels thereafter is consistent with widespread early transmission in Accra. Seroprevalence estimates exceeded those reported through routine surveillance and were also higher than those observed in a PCR-based contact-tracing investigation and a community serosurvey conducted in markets, malls, and lorry stations between July 2020 and February 2021^15,22^. This comparison is informative because contact tracing actively identifies infections and is therefore more sensitive than passive reporting, whereas PCR testing detects only recent infections within the narrow window of viral RNA detectability. In contrast, anti–SARS CoV-2 IgG reflects cumulative exposure over months and captures both recent and prior infections, particularly asymptomatic cases that were unlikely to be identified by RT-PCR or rapid antigen testing. In the context of negligible to low vaccination coverage during most of the study period (~ 1.2% − 13% fully vaccinated (2 doses) from June 2021 to end of January 2022)^23–25^ and very high SARS CoV-2 asymptomatic rates (> 45%)^26,27^, these findings indicate that community transmission was substantially higher than suggested even by enhanced surveillance. These findings are consistent with widespread infection-derived exposure^28^. After wave 2, seroprevalence plateaued at near-universal levels. Antibody concentrations increased markedly through wave 3 but stabilised between waves 3 and 4, likely reflecting an equilibrium between antibody waning and ongoing antigenic re-exposure, consistent with findings from longitudinal population-level SARS CoV-2 serological studies^29^.

Although seropositivity converged across age groups, the timing of exposure differed markedly. Adults aged ≥ 20 years reached widespread seropositivity by wave 2, whereas children and adolescents accumulated exposure more gradually and reached comparable levels only in later waves. This delayed and more gradual increase in seroprevalence among younger age groups is consistent with reduced early social mixing during periods of school closure and movement restrictions. Age-related differences in infection severity, antibody response magnitude, and the likelihood of detectable seroconversion have been proposed as alternative explanations; however, several observations from our data argue against these as primary drivers. First, wave 1 seroprevalence was broadly comparable across most age groups, including children aged 0–9 and 10–19 years, indicating that the assay reliably detected antibody responses in younger individuals when exposure had occurred. Second, among seropositive individuals, anti-RBD IgG titres in the < 20 years group were comparable to those in working-age adults in wave 1 (GMT 148 vs. 161 BAU/mL; *p* = 0.865). Divergence emerged in subsequent waves as titres increased more rapidly in older age groups, consistent with differential cumulative exposure rather than an intrinsic deficit in antibody response magnitude in children. Third, the high population-level seroprevalence observed across all age groups, in the absence of a commensurate clinical case burden, indicates that mild and asymptomatic infection was the dominant pattern across the population rather than a pattern specific to younger age groups. Taken together, the lower and more delayed seroprevalence in younger age groups more plausibly reflects true differences in cumulative exposure, consistent with findings from multiple studies^25,30,31^.

Quantitative antibody titres provided additional insight once seroprevalence approached saturation. Antibody concentrations increased markedly through wave 3, consistent with repeated immune stimulation at the population level, in line with longitudinal studies demonstrating stepwise antibody shift in titres following repeated infection or exposure to antigenically distinct variants^32,33^.

To further characterise population-level shifts in antibody titre distributions, we calculated the proportions of samples in each wave whose anti-RBD IgG concentration exceeded ≥ 4-fold and ≥ 8-fold multiples of the age-specific wave 1 GMT. We acknowledge that in a repeated cross-sectional design without paired individual measurements, these thresholds cannot be interpreted as within-individual fold-rises. Instead, they serve as population-level indicators of antibody titre accumulation across successive waves — reflecting distributional shifts rather than individual immune trajectories. Between-individual variation in baseline antibody levels represents a source of uncertainty in this approach. however, the progressive increase in the proportion of samples exceeding these thresholds across successive epidemic waves, the consistent age-stratified patterns observed, and the temporal alignment with documented SARS CoV-2 variant turnover in Ghana support the biological plausibility of the findings^19–21^. Collectively, these observations suggest a biological signal consistent with cumulative antigenic exposure rather than random variation. Experimental re-exposure models and longitudinal studies demonstrate that secondary antigenic exposure is typically accompanied by multi-fold antibody increases, supporting the epidemiological use of such distributional metrics in cross-sectional surveillance settings^32,33^.

A substantial epidemic wave occurred in January 2022 (wave 4; Fig. 1A) despite near universal seropositivity (98.5%) and high antibody concentrations. The minimal increase in seroprevalence between waves 3 and 4 (96.6% to 98.5%), together with persistently elevated antibody titres (geometric mean 625 to 584 BAU/mL), suggests that transmission was largely driven by infections among previously exposed individuals rather than primary infections in the small remaining seronegative population. This interpretation aligns with the emergence of Omicron BA.1/BA.2 during this period (Fig. 1B), variants known to partially evade infection-derived immunity^34–36^. While some new infections likely occurred among the remaining seronegative individuals—predominantly younger children—the majority of transmission plausibly involved re-exposure of seropositive adults, who had already reached ≥ 97% seropositivity by wave 2. These findings indicate that very high population-level seroprevalence does not prevent subsequent epidemic waves when antigenically distinct variants emerge, although prior exposure likely continued to mitigate severe disease.

Age-stratified titre distribution analyses revealed distinct cumulative exposure trends. Higher proportions of older adults showing concentrations substantially exceeding wave 1 baseline levels indicate earlier and more frequent antigenic stimulation across successive waves. In contrast, delayed and less frequent accumulation among younger age groups mirrors their later rise in seropositivity and lower overall antibody concentrations. Importantly, these findings do not imply uniform re-exposure events across all age groups but are most plausibly explained by variation in immune histories shaped by contact patterns, epidemic timing, and circulating variants^25,37–39^. Together, these findings highlight the value of quantitative serological measures for interpreting exposure dynamics in high-transmission settings and are consistent with observations from a comparable study in the United States^40^.

Residual clinical sera provided a pragmatic approach for population-level serosurveillance during the pandemic. However, detailed clinical indications and within-hospital collection-setting data were unavailable, limiting granular assessment of sampling representativeness. Nevertheless, analyses stratified by facility demonstrated comparable temporal increases in seroprevalence across epidemic waves, with differences largely explained by variation in age structure between sites, particularly the younger catchment population at the paediatric hospital. Multivariable analyses further identified epidemic wave progression, rather than collection site, as the principal determinant of seropositivity (Supplementary Table S5 – S8). Nonetheless, as all collection sites were hospital-based, the study population may over-represent individuals with greater healthcare contact. However, evidence from high-transmission settings indicates that seroprevalence estimates from such samples are broadly comparable to those from household surveys^41–44^.

In addition, sampling was conducted during the peak and declining phases of epidemic waves rather than after complete wave resolution, potentially underestimating cumulative exposure. Furthermore, vaccination histories for individual samples were unavailable because specimens were anonymised. However, vaccination coverage remained low during much of the study period, and no significant differences in anti-RBD IgG titres were observed between adults aged ≥ 60 years and those aged 20–59 years from wave 2 onward, suggesting limited impact of early vaccine rollout on the overall serological patterns observed.

## Conclusion

Seroprevalence data and SARS CoV-2–specific antibody measurements from August 2020 to January 2022 indicate intense and repeated transmission in Accra, resulting in near-universal exposure of the adult population by May 2021. Children and adolescents showed a slower, more gradual increase in both seroprevalence and antibody concentrations. Geometric mean antibody titres peaked between August and November 2021 and stabilised thereafter, consistent with equilibrium between waning and antigenic exposure, with no further increase by January 2022.

## Electronic Supplementary Material

Below is the link to the electronic supplementary material.

Supplementary Material 1

## Data Availability

The datasets generated and/or analysed during the current study are not publicly available due to ethical and data protection restrictions, as they contain potentially identifiable patient information. De-identified data may be made available from the corresponding author on reasonable request and with permission from the Ghana Health Service Ethics Review Committee.

## Abbreviations

BAU: Binding antibody units
COVID-19: Coronavirus disease 2019
ELISA: Enzyme-linked immunosorbent assay
IgG: Immunoglobulin G
NP: Nucleoprotein
RBD: Receptor binding domain
SARS CoV-2: Severe acute respiratory syndrome coronavirus 2

## References

[1] Kobia, F. & Gitaka, J. COVID-19: Are Africa’s diagnostic challenges blunting response effectiveness? *AAS Open. Res.* **3**, 4. 10.12688/AASOPENRES.13061.1 (2020). 32399515 10.12688/aasopenres.13061.1 PMC7205356

[2] Rice, B. L. et al. Variation in SARS-CoV-2 outbreaks across sub-Saharan Africa. *Nat. Med.* **27**, 447–453. 10.1038/s41591-021-01234-8 (2021). 33531710 10.1038/s41591-021-01234-8 PMC8590469

[3] Chen, X. et al. Ratio of asymptomatic COVID-19 cases among ascertained SARS-CoV-2 infections in different regions and population groups in 2020: A systematic review and meta-analysis including 130 123 infections from 241 studies. *BMJ Open.* 11. 10.1136/BMJOPEN-2021-049752 (2021). 10.1136/bmjopen-2021-049752 PMC8655350 34876424

[4] Hajissa, K. et al. Seroprevalence of SARS-CoV-2 Antibodies in Africa: A Systematic Review and Meta-Analysis. *Int. J. Environ. Res. Public. Health*. **19**, 7257. 10.3390/IJERPH19127257/S1 (2022). 35742506 10.3390/ijerph19127257 PMC9223681

[5] Adebisi, Y. A., Rabe, A. & Lucero-Prisno, D. E. COVID-19 surveillance systems in African countries. *Health Promot Perspect.* **11**, 382. 10.34172/HPP.2021.49 (2021). 35079582 10.34172/hpp.2021.49 PMC8767077

[6] Koech, A. et al. SARS-CoV-2 seroprevalence in pregnant women in Kilifi, Kenya from March 2020 to March 2022. *Front. Public. Health*. **11**, 1292932. 10.3389/FPUBH.2023.1292932/BIBTEX (2023). 38169905 10.3389/fpubh.2023.1292932 PMC10760635

[7] Kenu, E., Frimpong, J. A. & Koram, K. A. Responding to the COVID-19 pandemic in Ghana. *Ghana. Med. J.* **54**, 72–73. 10.4314/GMJ.V54I2.1 (2020). 33536675 10.4314/gmj.v54i2.1 PMC7829051

[8] Ghana Health Service. COVID-19 Updates | Ghana. (2022). https://ghs.gov.gh/covid19/archive.php. Accessed 2 Apr 2025.

[9] Kenu, E. et al. Epidemiology of COVID-19 outbreak in Ghana, 2020. *Ghana. Med. J.* **54 4 Suppl**, 5–15. 10.4314/GMJ.V54I4S.3 (2020). 33976436 10.4314/gmj.v54i4s.3 PMC8087358

[10] Arnold, B. F., Scobie, H. M., Priest, J. W. & Lammie, P. J. Integrated Serologic Surveillance of Population Immunity and Disease Transmission - 24, Number 7—July 2018 - Emerging Infectious Diseases journal - CDC. *Emerg. Infect. Dis.* **24**, 1188–1194. 10.3201/EID2407.171928 (2018). 29912680 10.3201/eid2407.171928 PMC6038749

[11] Enyereibe, N. W. et al. SARS-CoV-2 seroprevalence and COVID-19 vaccination coverage in two states of Nigeria from a population based household survey. *Sci. Rep.* **15**, 1–12. 10.1038/S41598-025-14253-Z;SUBJMETA (2025). 40784905 10.1038/s41598-025-14253-z PMC12336329

[12] Owusu Donkor, I. et al. Modeling SARS-CoV-2 antibody seroprevalence and its determinants in Ghana: A nationally representative cross-sectional survey. *PLOS Global Public. Health*. **3**, e0001851. 10.1371/journal.pgph.0001851 (2023). 37145991 10.1371/journal.pgph.0001851 PMC10162519

[13] Mensah, B. A. et al. Population-based sero-epidemiological investigation of the dynamics of SARS-CoV-2 infections in the Greater Accra Region of Ghana. *Sci. Rep.* **12**, 21582. 10.1038/s41598-022-25598-0 (2022). 36517505 10.1038/s41598-022-25598-0 PMC9748398

[14] Partey, F. D. et al. Efficient transplacental transfer of SARS-CoV-2 antibodies between naturally exposed mothers and infants in Accra, Ghana. *Sci. Rep.* **14**, 10772. 10.1038/S41598-024-61496-3 (2024). 38730052 10.1038/s41598-024-61496-3 PMC11087586

[15] Quashie, P. K. et al. Trends of severe acute respiratory syndrome coronavirus 2 (SARS-CoV-2) antibody prevalence in selected regions across Ghana. *Wellcome Open. Res.* **6**, 173. 10.12688/WELLCOMEOPENRES.16890.1 (2021).

[16] 2021 Population and Housing Census - Ghana Statistical Service. Jul (2025). https://census2021.statsghana.gov.gh/. Accessed 5.

[17] Pappoe-Ashong, P. J. et al. Clinical performance of a dual-target SARS CoV-2 antibody assay using sera from Ghana. *BMC Infect. Dis.* **25**, 1552. 10.1186/s12879-025-12012-z (2025). 41225345 10.1186/s12879-025-12012-z PMC12613554

[18] Reiners, N. et al. Performance of a SARS CoV-2 antibody ELISA based on simultaneous measurement of antibodies against the viral nucleoprotein and receptor-binding domain. *Eur. J. Clin. Microbiol. Infect. Dis.* **40**, 2645–2649. 10.1007/s10096-021-04284-5 (2021). 34085159 10.1007/s10096-021-04284-5 PMC8175097

[19] Morang’a, C. M. et al. Genetic diversity of SARS-CoV-2 infections in Ghana from 2020–2021. *Nat. Commun.* **13**, 2494. 10.1038/s41467-022-30219-5 (2022). 35523782 10.1038/s41467-022-30219-5 PMC9076825

[20] Asante, I. A. et al. Detection of SARS-CoV-2 Variants Imported Through Land Borders at the Height of the COVID-19 Pandemic in Ghana, 2022. *Cureus* 16. 10.7759/CUREUS.68220 (2024). 10.7759/cureus.68220 PMC11439440 39347199

[21] Lomotey, E. S. et al. Evaluation of SARS-CoV-2 Seroprevalence and Variant Distribution During the Delta-Omicron Transmission Waves in Greater Accra, Ghana, 2021. *Viruses* 17. 10.3390/v17040487 (2025). 10.3390/v17040487 PMC12031444 40284930

[22] Kenu, E. et al. Community-Based Surveillance and Geographic Information System–Linked Contact Tracing in COVID-19 Case Identification, Ghana, March–June 2020. *Emerg. Infect. Dis.* **28**, S114–S120. 10.3201/EID2813.221068 (2022). 36502391 10.3201/eid2813.221068 PMC9745224

[23] COVID-19 vaccination in the WHO African. Region – 7 February 2022 | WHO | Regional Office for Africa. Jan (2026). https://www.afro.who.int/publications/covid-19-vaccination-who-african-region-7-february-2022. Accessed 7.

[24] Mathieu, E. et al. A global database of COVID-19 vaccinations. *Nat. Hum. Behav.* **5**, 947–953. 10.1038/s41562-021-01122-8 (2021). 33972767 10.1038/s41562-021-01122-8

[25] Ofori, S. K. et al. Transmission Dynamics of COVID-19 in Ghana and the Impact of Public Health Interventions. *Am. J. Trop. Med. Hyg.* **107**, 175–179. 10.4269/AJTMH.21-0718 (2022). 35605636 10.4269/ajtmh.21-0718 PMC9294683

[26] Owoo, C. et al. Sociodemographic and clinical characteristics of the first cohort of COVID-19 recoveries at two national treatment centres in Accra, Ghana. *Ghana. Med. J.* **54** (4 Suppl), 16–22. 10.4314/GMJ.V54I4S.4 (2020). 33976437 10.4314/gmj.v54i4s.4 PMC8087372

[27] Oduro-Mensah, E. et al. Clinical features of COVID-19 in Ghana: symptomatology, illness severity and comorbid non-communicable diseases. *Ghana. Med. J.* **54**10.4314/GMJ.V54I4S.5 (2020). 4 Suppl:23. 10.4314/gmj.v54i4s.5 PMC8087366 33976438

[28] Merkt, S. et al. Long-term monitoring of SARS-CoV-2 seroprevalence and variants in Ethiopia provides prediction for immunity and cross-immunity. *Nat. Commun. 2024*. **15**, 1. 10.1038/s41467-024-47556-2 (2024). 10.1038/s41467-024-47556-2 PMC11043357 38658564

[29] Nilles, E. J. et al. Convergence of SARS-CoV-2 spike antibody levels to a population immune setpoint. *EBioMedicine* **108**, 105319. 10.1016/j.ebiom.2024.105319 (2024). 39232463 10.1016/j.ebiom.2024.105319 PMC11404201

[30] Zhang, J. et al. Changes in contact patterns shape the dynamics of the COVID-19 outbreak in China. *Science* **368**, 1481–1486. 10.1126/SCIENCE.ABB8001 (2020). 32350060 10.1126/science.abb8001 PMC7199529

[31] Gobena, D. et al. Escalating spread of SARS-CoV-2 infection after school reopening among students in hotspot districts of Oromia Region in Ethiopia: Longitudinal study. *PLoS One*. 18. 10.1371/JOURNAL.PONE.0280801 (2023). 10.1371/journal.pone.0280801 PMC9897530 36735689

[32] Siddiqui, S. M. et al. Serological Markers of SARS-CoV-2 Reinfection. *mBio* 13. 10.1128/MBIO.02141-21 (2022). 10.1128/mbio.02141-21 PMC8787477 35073738

[33] Lindsey, K. M. et al. From first infection to reinfection: Comparing Nucleocapsid antibody kinetics in vaccinated and unvaccinated adults. *Vaccine* **62**, 127593. 10.1016/j.vaccine.2025.127593 (2025). 40812020 10.1016/j.vaccine.2025.127593 PMC13238270

[34] Pulliam, J. R. C. et al. Increased risk of SARS-CoV-2 reinfection associated with emergence of Omicron in South Africa. *Science* **376**, eabn4947. 10.1126/science.abn4947 (2022). 35289632 10.1126/science.abn4947 PMC8995029

[35] Dimeglio, C. et al. Antibody titers and breakthrough infections with Omicron SARS-CoV-2. *J. Infect.* **84**, e13–e15. 10.1016/j.jinf.2022.01.044 (2022). 35123960 10.1016/j.jinf.2022.01.044 PMC8812090

[36] Nguyen, N. N. et al. High rate of reinfection with the SARS-CoV-2 Omicron variant. *J. Infect.* **85**, 174–211. 10.1016/j.jinf.2022.04.034 (2022). 35472367 10.1016/j.jinf.2022.04.034 PMC9033627

[37] Bagala, I. et al. Seroprevalence of SARS-CoV-2 and risk factors for infection among children in Uganda: A serial cross-sectional study. *PLoS One*. 19. 10.1371/JOURNAL.PONE.0312554 (2024). 10.1371/journal.pone.0312554 PMC11665985 39715211

[38] Mikolajczyk, R. T., Akmatov, M. K., Rastin, S. & Kretzschmar, M. Social contacts of school children and the transmission of respiratory-spread pathogens. *Epidemiol. Infect.* **136**, 813–822. 10.1017/S0950268807009181 (2008). 17634160 10.1017/S0950268807009181 PMC2870867

[39] Mossong, J. et al. Social Contacts and Mixing Patterns Relevant to the Spread of Infectious Diseases. *PLoS Med.* **5**, e74. 10.1371/JOURNAL.PMED.0050074 (2008). 18366252 10.1371/journal.pmed.0050074 PMC2270306

[40] Bratcher, A. et al. Quantitative SARS-CoV-2 Spike Receptor-Binding Domain and Neutralizing Antibody Titers in Previously Infected Persons, United States, January 2021-February 2022. *Emerg. Infect. Dis.* **30**, 2352–2361. 10.3201/eid3011.240043 (2024). 39447163 10.3201/eid3011.240043 PMC11521179

[41] Bajema, K. L. et al. Comparison of Estimated Severe Acute Respiratory Syndrome Coronavirus 2 Seroprevalence Through Commercial Laboratory Residual Sera Testing and a Community Survey. *Clin. Infect. Dis.* **73**, E3120–E3123. 10.1093/CID/CIAA1804 (2021). 33300579 10.1093/cid/ciaa1804 PMC7799302

[42] Kao, S-Y-Z., Nycz, E., Benoit, T. J., Clarke, K. E. N. & Jones, J. M. Comparison of SARS-CoV-2 seroprevalence estimates between commercial lab serum specimens and blood donor specimens, United States, September-December 2021. *Microbiol. Spectr.* 12. 10.1128/SPECTRUM.00123-24 (2024). 10.1128/spectrum.00123-24 PMC11302068 38869287

[43] Nishigaki, A. et al. Comparison of Infection-Induced SARS-CoV-2 Seroprevalence Across Large-Scale Residual Samples From Blood Donors, Commercial Laboratories, and Health Checkups in Japan, 2023. *Open. Forum Infect. Dis.* 12. 10.1093/OFID/OFAF415 (2025). 10.1093/ofid/ofaf415 PMC12321519 40765712

[44] Tunheim, G. et al. Prevalence of antibodies against SARS-CoV-2 in the Norwegian population, August 2021. *Influenza Other Respir Viruses*. **16**, 1004–1013. 10.1111/IRV.13024 (2022). 35770841 10.1111/irv.13024 PMC9349429

