# Supplementary material for: SARS CoV-2 seroprevalence and rising antibody titres across successive pandemic waves in Accra, Ghana: August 2020 to January 2022
Source: Sci Rep. 2026 Jun 22;16:19334. doi: 10.1038/s41598-026-57566-3 (PMC13287567; doi:10.1038/s41598-026-57566-3)
Supplement: Supplementary file 1 — Supplementary Material 1 [file 41598_2026_57566_MOESM1_ESM.docx]

**Supplementary Table S1. Age-stratified anti–SARS CoV-2 IgG seroprevalence across four epidemic waves in Accra, Ghana**

| **Wave** | **Age group** | **N** | **Seropositive, n** | **Seroprevalence % (95% CI)** |
| --- | --- | --- | --- | --- |
| **Wave 1** | 0–9 years | 54 | 29 | 53.7 (40.6–66.4) |
|  | 10–19 years | 51 | 26 | 51.0 (37.2–64.7) |
|  | 20–29 years | 84 | 53 | 63.1 (52.3–72.8) |
|  | 30–39 years | 96 | 54 | 56.3 (46.1–65.9) |
|  | 40–49 years | 80 | 45 | 56.3 (45.1–66.9) |
|  | 50–59 years | 58 | 50 | 86.2 (75.3–92.9) |
|  | ≥60 years | 26 | 18 | 69.2 (49.2–84.0) |
|  | **Overall** | **499** | **291** | **58.3 (53.9–62.6)** |
| **Wave 2** | 0–9 years | 22 | 16 | 72.7 (50.9–87.3) |
|  | 10–19 years | 38 | 35 | 92.1 (79.2–97.3) |
|  | 20–29 years | 37 | 36 | 97.3 (86.2–99.5) |
|  | 30–39 years | 38 | 38 | 100.0 (90.8–100) |
|  | 40–49 years | 38 | 34 | 89.5 (75.9–95.8) |
|  | 50–59 years | 18 | 18 | 100.0 (82.4–100) |
|  | ≥60 years | 12 | 12 | 100.0 (75.8–100) |
|  | **Overall** | **203** | **185** | **91.1 (86.4–94.3)** |
| **Wave 3** | 0–9 years | 35 | 31 | 88.6 (74.0–95.6) |
|  | 10–19 years | 28 | 28 | 100.0 (88.1–100) |
|  | 20–29 years | 37 | 37 | 100.0 (90.5–100) |
|  | 30–39 years | 37 | 37 | 100.0 (90.5–100) |
|  | 40–49 years | 38 | 37 | 97.4 (86.5–99.5) |
|  | 50–59 years | 26 | 26 | 100.0 (87.1–100) |
|  | ≥60 years | 13 | 13 | 100.0 (77.2–100) |
|  | **Overall** | **203** | **196** | **96.6 (93.1–98.3)** |
| **Wave 4** | 0–9 years | 46 | 45 | 97.8 (88.5–99.6) |
|  | 10–19 years | 26 | 26 | 100.0 (87.1–100) |
|  | 20–29 years | 34 | 34 | 100.0 (90.0–100) |
|  | 30–39 years | 36 | 36 | 100.0 (90.3–100) |
|  | 40–49 years | 19 | 19 | 100.0 (83.2–100) |
|  | 50–59 years | 18 | 18 | 100.0 (82.4–100) |
|  | ≥60 years | 11 | 11 | 100.0 (74.1–100) |
|  | **Overall** | **193** | **190** | **98.5 (95.5–99.5)** |

**Supplementary Table S2. Age-Stratified SARS CoV-2 Antibody Geometric Mean Titres (GMTs) by Wave**

| **Wave** | **Age Group** | **N** | **GMT^1^** | **95% CI** |
| --- | --- | --- | --- | --- |
| Wave 1 | <20 | 53 | 148 | 122.0–180.1 |
|  | 20–59 | 156 | 161 | 140.8–184.1 |
|  | ≥60 | 17 | 85 | 53.7–133.5 |
| Wave 2 | <20 | 67 | 169 | 132.9–215.0 |
|  | 20–59 | 106 | 392 | 308.5–498.6 |
|  | ≥60 | 12 | 525 | 254.3–1082.3 |
| Wave 3 | <20 | 55 | 261 | 201.3–339.6 |
|  | 20–59 | 128 | 728 | 598.2–885.0 |
|  | ≥60 | 13 | 1157 | 490.1–2732.5 |
| Wave 4 | <20 | 67 | 369 | 268.0–508.2 |
|  | 20–59 | 101 | 718 | 562.0–917.3 |
|  | ≥60 | 22 | 773 | 445.4–1340.0 |
| ^1^Includes seropositive participants only (anti-RBD IgG ≥32 BAU/mL) at each wave. | | | | |

**Supplementary Table S3. Pairwise Comparisons of Anti-RBD IgG GMTs Between Age Groups Across Pandemic Waves**

| **Wave** | **Comparison** | **Group 1 (GMT, 95% CI)** | **Group 2 (GMT, 95% CI)** | **p-value** |
| --- | --- | --- | --- | --- |
| Wave 1 | <20 vs 20–59 | 148 (122.0–180.1) | 161 (140.8–184.1) | 0.8654 |
|  | <20 vs ≥60 | 148 (122.0–180.1) | 85 (53.7–133.5) | 0.0122* |
|  | 20–59 vs ≥60 | 161 (140.8–184.1) | 85 (53.7–133.5) | 0.0037* |
| Wave 2 | <20 vs 20–59 | 169 (132.9–215.0) | 392 (308.5–498.6) | <0.001* |
|  | <20 vs ≥60 | 169 (132.9–215.0) | 525 (254.3–1082.3) | 0.0032* |
|  | 20–59 vs ≥60 | 392 (308.5–498.6) | 525 (254.3–1082.3) | 0.4359 |
| Wave 3 | <20 vs 20–59 | 261 (201.3–339.6) | 728 (598.2–885.0) | <0.001* |
|  | <20 vs ≥60 | 261 (201.3–339.6) | 1157 (490.1–2732.5) | 0.0003* |
|  | 20–59 vs ≥60 | 728 (598.2–885.0) | 1157 (490.1–2732.5) | 0.4227 |
| Wave 4 | <20 vs 20–59 | 369 (268.0–508.2) | 718 (562.0–917.3) | 0.0041* |
|  | <20 vs ≥60 | 369 (268.0–508.2) | 773 (445.4–1340.0) | 0.0320* |
|  | 20–59 vs ≥60 | 718 (562.0–917.3) | 773 (445.4–1340.0) | 0.8586 |
| GMT values are presented as geometric mean titres (95% CI). Group 1 and Group 2 correspond to the first- and second-named age groups in the Comparison column, respectively. *Statistically significant (p < 0.05). Pairwise comparisons were performed using the Mann–Whitney U test within each wave*.* | | | | |

**Supplementary Table S4:** **Statistically Significant Pairwise Comparisons of High Antibody Responses Across Age Groups**

| **≥4× GMT** | | | **≥8× GMT** | | |
| --- | --- | --- | --- | --- | --- |
| **Age-group**  **comparison** | **Proportion of responders (%)** | **Raw**  **p-value** | **Age-group**  **comparison** | **Proportion of responders (%)** | **Raw**  **p-value** |
| *0–9 vs 30–39*** | *3.1 vs 37.5* | *0.0012* | *0–9 vs ≥60*** | *0.0 vs 41.7* | *0.0007* |
| *0–9 vs 40–49*** | *3.1 vs 41.2* | *0.0014* | **0–9 vs 20–29***** | **0.0 vs 31.6** | **<0.0001** |
| *0–9 vs 50–59*** | *3.1 vs 58.3* | *0.0001* | **0–9 vs 40–49***** | **0.0 vs 28.6** | **0.0011** |
| *0–9 vs ≥60*** | *3.1 vs 75.0* | *<0.0001* | **0–9 vs 50–59***** | **0.0 vs 53.8** | **<0.0001** |
| *10–19 vs ≥60*** | *14.3 vs 75.0* | *0.0002* | **0–9 vs ≥60***** | **0.0 vs 69.2** | **<0.0001** |
| **0–9 vs 10–19***** | **2.6 vs 37.5** | **0.0016** | **30–39 vs 50–59***** | **8.1 vs 53.8** | **0.0014** |
| **0–9 vs 20–29***** | **2.6 vs 64.9** | **<0.0001** | **30–39 vs ≥60***** | **8.1 vs 69.2** | **<0.0001** |
| **0–9 vs 30–39***** | **2.6 vs 59.5** | **<0.0001** | 0–9 vs 50–59**** | 25.0 vs 72.2 | 0.0012 |
| **0–9 vs 40–49***** | **2.6 vs 57.1** | **<0.0001** | 10–19 vs 50–59**** | 17.4 vs 72.2 | 0.0011 |
| **0–9 vs 50–59***** | **2.6 vs 69.2** | **<0.0001** | 20–29 vs 50–59**** | 11.8 vs 72.2 | <0.0001 |
| **0–9 vs ≥60***** | **2.6 vs 76.9** | **<0.0001** | 20–29 vs ≥60**** | 11.8 vs 54.5 | 0.0008 |
|  |  |  | 30–39 vs 50–59**** | 16.7 vs 72.2 | 0.0002 |
|  |  |  | 40–49 vs 50–59**** | 15.8 vs 72.2 | 0.0008 |
| Pairwise comparisons of age-group differences in the proportion of participants achieving ≥4× and ≥8× increase in anti–SARS CoV-2 RBD IgG titres relative to baseline geometric mean titres (GMTs) across pandemic waves. Statistical significance was assessed using Fisher's exact tests with Bonferroni correction for multiple comparisons. Raw p-values are reported. To control for 21 pairwise comparisons per wave and threshold, a Bonferroni-adjusted significance threshold of p < 0.00238 (α = 0.05/21) was applied. Comparisons not reaching this threshold are not shown. Wave indicators: ** = Wave 2, *** = Wave 3, **** = Wave 4. | | | | | |

**Supplementary Table S5. Site-level SARS CoV-2 seroprevalence across epidemic waves (with 95% CIs)**

| **Wave** | **Site** | **Median age, years (IQR)** | **Total (N)** | **Seropositive (n)** | **Seroprevalence % (95% CI)** |
| --- | --- | --- | --- | --- | --- |
| Wave 1 | PMLH | 5 (1.5–12.0) | 181 | 93 | 51.4 (44.2–58.6) |
|  | GARH | 37 (28.8–46.0) | 153 | 89 | 58.2 (50.3–65.7) |
|  | UH | 27 (21.0–40.0) | 165 | 109 | 66.1 (58.5–73.0) |
|  | Overall | — | 499 | 291 | 58.3 (54.0–62.5) |
| Wave 2 | PMLH | 4 (1.4–10.0) | 54 | 42 | 77.8 (64.4–87.3) |
|  | GARH | 29 (23.3–42.0) | 79 | 77 | 97.5 (91.2–99.3) |
|  | UH | 30 (18.0–40.0) | 70 | 66 | 94.3 (86.0–97.8) |
|  | Overall | — | 203 | 185 | 91.1 (86.4–94.3) |
| Wave 3 | PMLH | 3 (1.7–6.0) | 53 | 46 | 86.8 (74.7–93.6) |
|  | GARH | 33.5 (26.0–44.0) | 120 | 120 | 100.0 (96.9–100) |
|  | UH | 26 (22.0–33.8) | 30 | 30 | 100.0 (88.7–100) |
|  | Overall | — | 203 | 196 | 96.6 (93.1–98.4) |
| Wave 4 | PMLH | 4.4 (2.0–8.0) | 52 | 51 | 98.1 (89.9–99.7) |
|  | GARH | 42 (33.0–58.5) | 90 | 88 | 97.8 (92.3–99.4) |
|  | UH | 26 (20.0–30.0) | 51 | 51 | 100.0 (93.0–100) |
|  | Overall | — | 193 | 190 | 98.4 (95.5–99.5) |

**Supplementary Table S6. Pairwise comparisons of SARS CoV-2 seroprevalence between study sites across four epidemic waves using Fisher’s exact test with Bonferroni correction**

| **Wave** | **Comparison** | **Seroprevalence A** | **Seroprevalence B** | **Raw p** | **Adjusted p** | **Significance** |
| --- | --- | --- | --- | --- | --- | --- |
| Wave 1 | PMLH vs GARH | 51.4% | 58.2% | 0.227 | 1.000 | ns |
|  | GARH vs UH | 58.2% | 66.1% | 0.165 | 1.000 | ns |
|  | UH vs PMLH | 66.1% | 51.4% | 0.006 | 0.077 | ns† |
| Wave 2 | PMLH vs GARH | 77.8% | 97.5% | 0.0004 | 0.005 | ** |
|  | GARH vs UH | 97.5% | 94.3% | 0.420 | 1.000 | ns |
|  | UH vs PMLH | 94.3% | 77.8% | 0.013 | 0.155 | ns |
| Wave 3 | PMLH vs GARH | 86.8% | 100% | 0.0002 | 0.002 | ** |
|  | GARH vs UH | 100% | 100% | 1.000 | 1.000 | ns |
|  | UH vs PMLH | 100% | 86.8% | 0.045 | 0.542 | ns |
| Wave 4 | PMLH vs GARH | 98.1% | 97.8% | 1.000 | 1.000 | ns |
|  | GARH vs UH | 97.8% | 100% | 0.535 | 1.000 | ns |
|  | UH vs PMLH | 100% | 98.1% | 1.000 | 1.000 | ns |
| Pairwise comparisons of seroprevalence were performed between study sites within each epidemic wave using two-sided Fisher’s exact tests. P-values were adjusted for multiple comparisons using the Bonferroni correction (k = 12 total comparisons). Seroprevalence values represent the proportion of anti-RBD IgG seropositive samples at each site within the specified wave. PMLH = Princess Marie Louise Children’s Hospital; GARH = Greater Accra Regional Hospital; UH = Ussher Hospital. Statistical significance was defined as adjusted p < 0.05. Significance codes: ns, not significant; *p < 0.05; **p < 0.01. | | | | | | |

**Supplementary Table S7. Multivariable logistic regression: predictors of SARS CoV-2 seropositivity**

| **Predictor** | **β** | **SE** | **z** | **OR** | **95% CI** | **p-value** |
| --- | --- | --- | --- | --- | --- | --- |
| Intercept | −0.233 | 0.150 | −1.55 | 0.79 | 0.59–1.06 | 0.121 |
| Age (per year) | 0.013 | 0.007 | 1.93 | 1.013 | 1.000–1.027 | 0.053 |
| Wave 2 vs Wave 1 | 2.000 | 0.266 | 7.51 | 7.39 | 4.38–12.46 | **<0.001** |
| Wave 3 vs Wave 1 | 3.043 | 0.400 | 7.62 | 20.97 | 9.59–45.87 | **<0.001** |
| Wave 4 vs Wave 1 | 3.811 | 0.590 | 6.46 | 45.22 | 14.22–143.83 | **<0.001** |
| GARH vs PMLH | 0.237 | 0.288 | 0.82 | 1.27 | 0.72–2.23 | 0.410 |
| UH vs PMLH | 0.537 | 0.258 | 2.08 | 1.71 | 1.03–2.83 | **0.038** |
| Multivariable logistic regression was performed to identify predictors of SARS CoV-2 seropositivity. The dependent variable was anti-RBD IgG serostatus (seropositive vs seronegative). Predictor variables included age (continuous, per year increase), epidemic wave, and sampling facility. Wave 1 and Princess Marie Louise Children’s Hospital (PMLH) were used as reference categories. Odds ratios (ORs) with 95% confidence intervals (CIs) are shown. GARH = Greater Accra Regional Hospital; UH = Ussher Hospital. Statistically significant associations (p < 0.05) are shown in bold. | | | | | | |

**Supplementary Table S8. Model fit statistics for multivariable logistic regression models predicting SARS CoV-2 seropositivity.**

| **Statistic** | **Value** |
| --- | --- |
| Observations | 1,098 |
| AIC — Model 1 (age only) | 1122.40 |
| AIC — Model 2 (age + wave) | 882.83 |
| AIC — Model 3 (age + wave + cohort) | 881.83 |
| ΔAIC (Model 2 vs Model 3) | 1.0 |
| AUC (Model 3) | 0.816 |
| Model performance was assessed using Akaike Information Criterion (AIC) and area under the receiver operating characteristic curve (AUC). Lower AIC values indicate improved model fit after accounting for model complexity. Model 1 included age only; Model 2 included age and epidemic wave; Model 3 additionally included sampling facility. AUC values closer to 1 indicate better discrimination between seropositive and seronegative samples. | |
